# Antegrade double-J stenting as an alternative to the retrograde approach: experience of the first 150 cases at a single center in Brazil

**DOI:** 10.1590/0100-3984.2020.0131

**Published:** 2021

**Authors:** Renata Motta Grubert, Carlos Egydio Ferri do Carmo, Reinaldo Santos Morais Neto, Tiago Kojun Tibana, Rômulo Florêncio Tristão Santos, Edson Marchiori, Thiago Franchi Nunes

**Affiliations:** 1 Hospital Universitário Maria Aparecida Pedrossian da Universidade Federal de Mato Grosso do Sul (HUMAP-UFMS), Campo Grande, MS, Brazil.; 2 Universidade Federal do Rio de Janeiro (UFRJ), Rio de Janeiro, RJ, Brazil.

**Keywords:** Urinary catheterization/instrumentation, Stents, Ureteral obstruction, Radiology, interventional, Urologic neoplasms, Cateterismo urinário/instrumentação, *Stents*, Obstrução ureteral, Radiologia intervencionista, Neoplasias urológicas

## Abstract

**Objective:**

To present our clinical experience with percutaneous antegrade ureteral stenting.

**Materials and Methods:**

This was a single-center retrospective study in which we reviewed the electronic medical records of patients who underwent percutaneous image-guided antegrade ureteral stenting between January 2016 and August 2020. We evaluated 90 patients (48 men). The mean age was 61.4 ± 15 years (range, 30-94 years). Patients were divided into two main groups: those with malignant neoplasms; and those with non-neoplastic disease. Technical and clinical success of the procedure were defined, respectively, as maintenance of the patency of the urinary tract, with a reduction in the degree of hydronephrosis, and as a reduction in the level of nitrogenous waste. Postprocedural complications were categorized as major or minor according to the CIRSE classification.

**Results:**

The study sample comprised 150 antegrade stenting procedures performed in 90 patients, most of whom had previously undergone retrograde stenting that was unsuccessful. The stenting was bilateral in 60 patients and unilateral in 30. Technical success was achieved in 143 (95.3%) of the procedures, whereas seven procedures (4.6%) were unsuccessful. Failed procedures were characterized by inability to place a stent or migration of a stent after its placement. Complications occurred in 12 (8.0%) of the procedures. Of those 12 complications, two were classified as major (bleeding) and 10 were classified as minor (lumbar pain or infection). The most common techniques used were the over-the-wire technique and the modified technique (in 58.0% and 42.0% of the cases, respectively). In seven cases (4.7%), a nephrostomy tube was inserted.

**Conclusion:**

Percutaneous antegrade ureteral stenting is a safe, effective method for the management of ureteral injuries and obstructions, due to malignant or benign causes, when the retrograde approach has failed.

## Abstract (pt)

**Objetivo:**

Apresentar os resultados clínicos de 150 casos de inserção anterógrada de cateter duplo J.

Materiais e Métodos: Foram revisados os prontuários eletrônicos de pacientes submetidos a inserção percutânea de cateter duplo J guiada por imagem entre janeiro de 2016 e agosto de 2020. Um total de 90 pacientes (48 homens e 42 mulheres; faixa etária, 30-94 anos; idade média, 61,4 ± 15 anos) foi incluído no estudo. Os pacientes foram classificados em dois grupos principais: neoplasia maligna e doença não neoplásica. O sucesso técnico e clínico do procedimento foi definido como a manutenção da perviedade da via urinária com redução do grau de hidronefrose e redução dos níveis das escórias nitrogenadas. As complicações pós-procedimento foram classificadas em maiores e menores, de acordo com o sistema de classificação CIRSE.

**Resultados:**

Foram realizados 150 procedimentos (90 pacientes) no período, sendo bilateral em 60 pacientes e unilateral em 30. Houve sucesso técnico em 143 casos (95,3%) e falhas em sete (4,7%), caracterizadas por migração e não progressão do cateter. Nossas taxas de complicações foram de 8,0% (12 casos), sendo dois maiores (sangramento) e 10 menores (principalmente dor lombar). As técnicas mais utilizadas para a inserção foram *over the wire* (58,0%) e modificada (42,0%). Em sete pacientes (4,7%) foi realizada nefrostomia percutânea.

**Conclusão:**

A inserção anterógrada do cateter duplo J é um método seguro e eficaz para o tratamento de obstruções uretéricas devidas a causas e lesões malignas e benignas, quando há uma falha na abordagem cistoscópica (retrógrada).

## INTRODUCTION

Due to their anatomical relationships with the surrounding organs and their long, narrow structure, the ureters are often affected by benign or malignant diseases, resulting in the interruption of urinary drainage. In most cases, the cause of ureteral obstruction is malignant, mainly due to pelvic tumors; in such cases, drainage of the urinary tract presents a higher risk of failure when the retrograde approach is used^**(1-3)**^. Chitale et al.^**(1)**^ retrospectively analyzed the success rates of insertion of a double-J (pigtail) stent (DJ stenting) for decompression of the urinary tract in cases of obstruction due to malignancy, comparing the antegrade percutaneous approach and the retrograde cystoscopic approach. The authors found that, over a two-year period, the antegrade approach had a success rate of 98%, with minimal morbidity, compared with only 21% for the retrograde approach.

Percutaneous nephrostomy is commonly used in order to relieve hydronephrosis, especially when the retrograde approach fails. The disadvantages of nephrostomy tubes with external drainage are the risk of infection^**(3)**^ and dislodgment of the drain^**(4)**^. In addition, patients may experience significant discomfort after the insertion of a nephrostomy tube. In contrast, double-J stents should be used preferentially for ureteral obstructions that require prolonged treatment.

Antegrade DJ stenting is performed by interventional radiologists under image guidance (by ultrasound, fluoroscopy, or both), having been widely described and indicated mainly for cases in which there is technical failure of the retrograde/cystoscopic approach^**(5-9)**^. Antegrade DJ stenting has been shown to be safe and effective in patients with ureteral obstruction^**(7)**^, with a good cost-benefit ratio when compared with percutaneous nephrostomy^**(7)**^. In most cases, antegrade DJ stenting is performed under light sedation with local anesthesia, which allows it to be performed in patients with serious clinical conditions that prevent them from undergoing deeper sedation (general anesthesia).

The objective of this study was to present the clinical outcomes, including technical success and complications, of antegrade DJ stenting. To that end, we evaluated the first 150 cases in which the procedure was performed at an interventional radiology research center in Brazil.

## MATERIALS AND METHODS

This study was approved by the local committee for ethics in research and in the management of teaching and research. Because of the retrospective nature of the study, the requirement for informed consent was waived.

Data collections and records were obtained, retrospectively, from the electronic medical records of patients who underwent antegrade DJ stenting between January 2016 and August 2020. We anonymized the information as numerical data, thus ensuring patient confidentiality. All of the patients included had been referred to the interventional radiology department for the treatment of ureteral obstruction from the oncology, nephrology or urology clinic. The urology clinic referred patients to the interventional radiology department if the retrograde approach failed or if the urology ward was full.

The study sample comprised 150 antegrade stenting procedures performed in 90 patients (48 men). The mean age was 61.4 ± 15 years (range, 30-94 years). Of those 150 procedures, 141 (94%) were performed after unsuccessful retrograde attempts, the remainder being performed for the treatment of a urinary fistula (two procedures), calculi in the distal ureter (two procedures), and migration of a ureteral double-J stent (five procedures).

**Figure 1:**
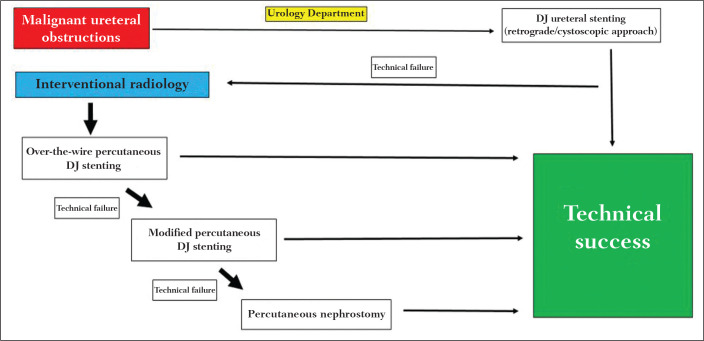
Clinic flow chart for the routine practice in patients with ureteral obstruction due to malignancy.

Demographic data, indications for the procedure, technical details of the procedure and post-procedure complications were collected from the relevant records, retrospectively. Patients were divided into two main groups: those with malignant neoplasms; and those with non-neoplastic disease. At our institution, we follow a flow chart for the management of ureteral obstruction caused by malignancy (Figure 1). Antegrade DJ stenting was performed in cases of ureteral obstruction only after a multidisciplinary discussion, mainly involving urologists, nephrologists, clinical oncologists, and interventional radiologists.

### Technical description

#### Over-the-wire

Percutaneous access to the renal collecting system is usually achieved with the patient in the supine position, with ultrasound guidance and an echogenic needle, thus allowing the visualization of the insertion from the skin to the renal calyx, preferably through the middle calyx, which offers easier access to the ureteropelvic junction, or through the inferior calyx, oriented posterolaterally, which provides a puncture route that is safe and relatively avascular, in order to minimize complications such as bleeding and pneumothorax. In cases of mild dilatation of the collecting system, the use of the coaxial technique with a micropuncture kit is preferred. Antegrade pyelography with iodinated contrast injection and fluoroscopic visualization of the anatomy of the collecting system is then performed. Once access has been established, a hydrophilic guidewire and a 6 Fr diagnostic catheter are introduced under fluoroscopy through the collecting system into the bladder. The diagnostic catheter is then removed from the bladder and the double-J stent is advanced along the hydrophilic or rigid guidewire.

#### Modified technique

Antegrade pyelography is performed after injection of nonionic iodinated contrast (350 mg I/mL) and fluoroscopic visualization of the anatomy of the collecting system, with immediate decompression after proper positioning of the needle. A 6 Fr introducer is placed at the ureteropelvic junction by the Seldinger technique. A 0.035’’ hydrophilic guidewire and a 5 Fr diagnostic catheter are introduced past the point of obstruction, and the catheter is positioned within the bladder. The hydrophilic guidewire is removed, and a 0.035’’ J-tipped Teflon-coated guidewire is positioned within the bladder. The 5 Fr catheter is removed, and a 6 Fr × 45 cm introducer sheath is put in place. The double-J stent is passed through the introducer sheath, with or without the Teflon-coated guidewire, and is then advanced, with the aid of the sheath dilator, until its lower end is within the bladder and the proper anchoring of the pigtail is confirmed by fluoroscopy. The introducer sheath is then retracted over the dilator (i.e., via “pullback”) until the latter is within the renal pelvis. Finally, the proximal (renal) end of the double-J stent is advanced with the aid of a dilator, for proper positioning within the renal collecting system.

#### Percutaneous nephrostomy

In all cases, ultrasound was performed prior to the procedure, in order to determine the nature and location of the obstruction. The minimum dilation of the renal pelvis was 20 mm. In all cases, a 10 Fr pigtail catheter was used. A 22G Chiba needle was inserted posterolaterally into the renal collecting system, through a renal calyx, under ultrasound and fluoroscopic guidance. Once the needle was in the collection system, urine was aspirated for microscopic analysis, after which contrast media was injected to identify the anatomy and a hydrophilic guidewire was inserted into the proximal ureter to guarantee access. This hydrophilic guidewire was then replaced with a rigid guidewire. The tract was expanded to 8 Fr and then to 10 Fr. The nephrostomy tube was then placed in the desired position and connected to an external drainage bag.

#### Technical and clinical success

Technical success of the procedure was defined as maintenance of the patency of the urinary tract with a reduction in the degree of hydronephrosis, as confirmed on imaging (ultrasound or computed tomography), and clinical success was defined as a reduction in the level of nitrogenous waste, as confirmed during inpatient and outpatient follow-up. A reduction in pain was an auxiliary subjective parameter, given that it affects patient quality of life, although it was not a definitive criterion for technical success.

#### Complications

Complications were classified according to the Cardiovascular and Interventional Radiological Society of Europe classification system^**(10)**^. Thus, major complications were defined as those requiring hospitalization (after an outpatient procedure), as well as those resulting in an unplanned increase in the level of care, prolonged hospitalization (> 48 h), permanent adverse sequelae, or death. Minor complications were defined as those that did not result in sequelae, did not require treatment, or required additional treatment with a short hospital stay for observation (typically overnight).

#### Statistical analysis

The data were entered into a spreadsheet and exported to the SPSS Statistics software package, version 24.0 for Windows (IBM Corp., Armonk, NY, USA), for statistical analysis. We analyzed descriptive statistics such as standard deviation from the mean (minimum and maximum), as well as absolute and relative frequencies.

### RESULTS

On the basis of our review of the electronic medical records, we included a total of 150 procedures performed in 90 patients (Tables 1-5): 18 (12%) performed in patients with non-neoplastic disease; and 132 (88%) performed in patients with malignant neoplasms. Of the 132 patients in the neoplastic group, 73 were female (mean age, 66.2 years) and 59 were male (mean age, 61.5 years). All 150 procedures involved percutaneous antegrade insertion of a double-J stent. The stenting was bilateral in 60 patients and unilateral in 30.

**Table 1:** Causes of ureteral obstruction treated with antegrade DJ stenting.

Cause of ureteral obstruction	(N = 150)
Tumor, n (%)	132 (88.0)
Surgical complications, n (%)	10 (6.7)
Urolithiasis, n (%)	6 (4.0)
Unknown, n (%)	2 (1.3)

**Table 2:** Neoplastic causes of ureteral obstruction treated with antegrade DJ stenting.

Type of neoplasia	(N = 132)
Cervical tumor, n (%)	47 (35.6)
Prostate tumor, n (%)	32 (24.2)
Bladder tumor, n (%)	24 (18.2)
Ovarian tumor, n (%)	18 (13.6)
Colorectal neoplasia, n (%)	8 (6.1)
Retroperitoneal tumor, n (%)	3 (2.3)

**Table 3:** Comparison of antegrade DJ stenting success rates across studies.

Studies	Success, n (%)
Uthappa et al.^(17)^, N = 25	24 (96)
Chitale et al.^(1)^, N = 40	39 (98)
Harding^(18)^, N = 37	34 (92)
Mitty et al.^(19)^, N = 78	67 (85)
Kahriman et al.^(20)^, N = 654	639 (97.7)
Present study, N = 150	143 (95.3)

**Table 4:** Types of ureteral tortuosity.

Ureteral tortuosity	(N = 150)
None, n (%)	67 (44.7)
Z-shaped, n (%)	60 (40.0)
Corkscrew, n (%)	23 (15.3)

**Table 5:** Rates of major and minor complications of antegrade DJ stenting.

Complication	(N = 150)
Major, n (%)	
Perirenal hematoma	2 (1.3)
Minor, n (%)	
Dysuria	6 (4.0)
Backache	3 (2.0)
Pyelonephritis	1 (0.7)
Total, n (%)	12 (8.0)

Technical success was achieved in 143 (95.3%) of the 150 procedures, and seven (4.7%) of the procedures were classified as technical failures: because of stent migration, with unsatisfactory urinary drainage at 24 h after the procedure, in two cases; and because of inability to place the stent, due to a tumor affecting > 5 cm of the middle and distal ureter, in five cases. In all cases of failure, we opted for percutaneous nephrostomy.

**Figure 2:**
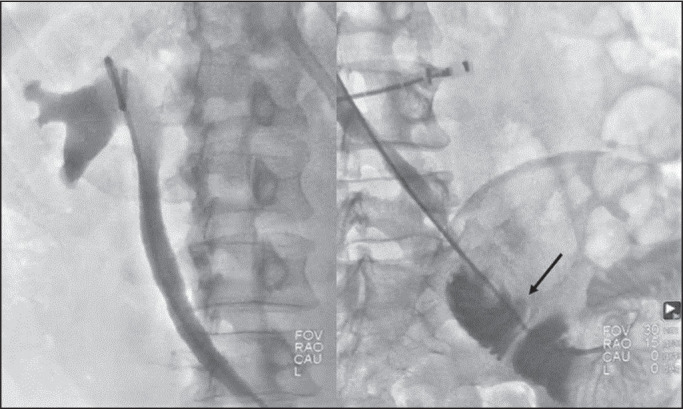
Antegrade DJ stenting with the over-the-wire technique in a patient with a neobladder (arrow) and benign distal stenosis.

**Figure 3:**
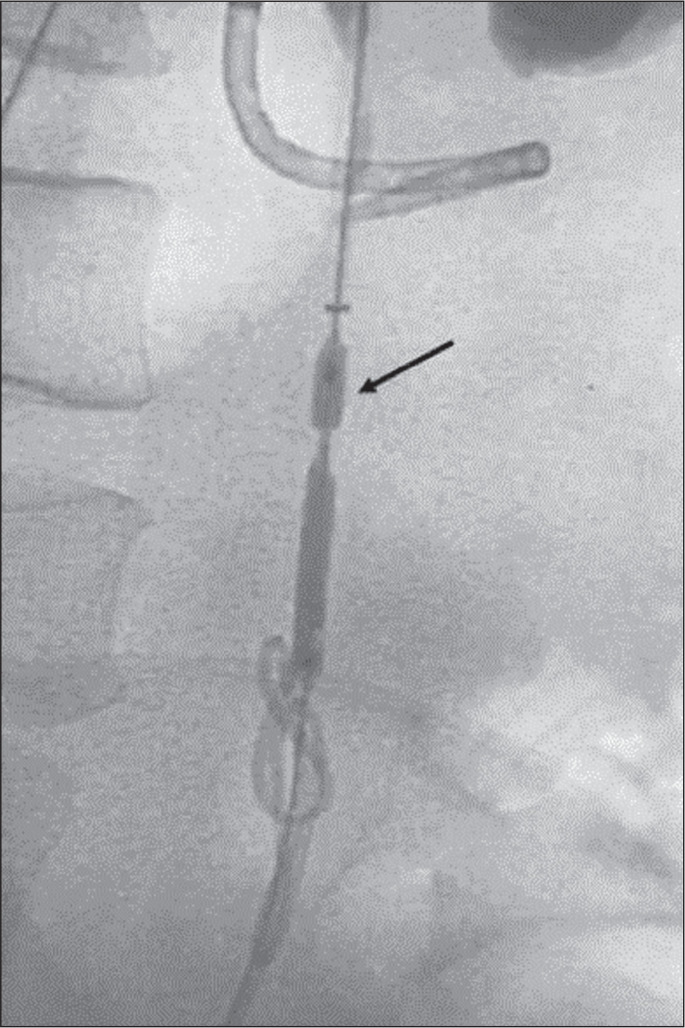
Patient with iatrogenic stenosis of the right proximal ureter, in whom antegrade (cystoscopic) DJ stenting was attempted without success. Because of the significant stenosis of the proximal ureter, dilation with a 4 × 80 mm balloon was performed. Note the balloon dilation (arrow) at the point of obstruction and the stent in place.

Of the 90 patients evaluated, 12 underwent antegrade DJ stenting more than once (twice, in three patients; three times, in two patients; and four or more times, in seven patients). In one patient, the distal end of the stent was inserted through the penile urethra, and in two patients, it was inserted up to a neobladder (Figure 2). In three cases, we performed balloon dilation (Figure 3), with technical success, shortly before the placement of the ureteral stent.

The most common causes of ureteral obstruction in the non-neoplastic group were iatrogenic post-ureterolithotripsy stenosis, urolithiasis, and stenosis of the ureteropelvic junction. The main indication in the neoplastic group was advanced neoplasia of the cervix or prostate.

Complications were observed in 12 (8%) of the 150 procedures evaluated, 10 being classified as minor complications and only two being classified as major complications. In the case of one of the major complications (perirenal hematoma), blood transfusion and prolongation of the hospital stay were necessary. Among the minor complications, the most common was low back pain. One patient developed pyelonephritis (a minor complication) and had a favorable evolution after being started on parenteral antibiotic therapy.

The over-the-wire technique was used in 83 (58%) of the 143 successful percutaneous DJ stenting procedures, whereas the modified technique was used in 60 (42%). In seven (4.7%) of the 150 procedures, the nephrostomy tube was left in place due to failure of the DJ stenting.

## DISCUSSION

In this retrospective study, we investigated the efficacy of antegrade DJ stenting for the treatment of ureteral obstruction caused by benign or malignant conditions. We found the antegrade DJ stenting technique to be a safe, effective method to use when the conventional (retrograde cystoscopic) approach fails.

**Figure 4:**
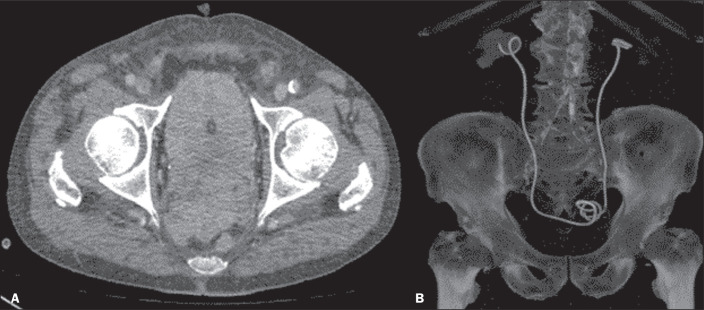
A: Advanced prostate cancer involving the rectum, pelvic muscles, and both ureteral ostia. Bilateral antegrade DJ stenting with the modified technique. B: Three-dimensional reconstruction demonstrating the proper positioning of both stents.

Success rates of 21-88% have been reported for retrograde (cystoscopic) DJ ureteral stenting in patients with ureteral obstruction due to malignancy^**(11-16)**^. However, to deal with technical difficulties that prevent the retrograde insertion of a DJ in such patients (Figure 4), antegrade DJ stenting is more appropriate. The technical success rate for DJ ureteral stenting in our sample (95.3%) compares favorably with the 85-98% reported by other authors^**(1,17-20)**^, as detailed in Table 3.

Chitale et al.^**(1)**^ retrospectively analyzed the success rates of DJ stenting for decompression of the urinary tract in cases of obstruction due to malignancy, comparing the antegrade percutaneous and retrograde approaches. The authors found that, over a two-year period, the antegrade approach had a success rate of 98%, with minimal morbidity, compared with only 21% for the retrograde cystoscopic approach. In that study, the sample comprised 65 patients, of whom 24 were initially treated with a retrograde approach (endoscopy) and 41 were initially treated with an antegrade approach (nephrostomy followed by insertion of a double-J stent). Among the 24 patients initially undergoing retrograde DJ stenting, technical failure of the procedure occurred in 19 (79%), who subsequently underwent antegrade DJ stenting, which was successful in 100% of those cases. Among the 41 patients initially undergoing antegrade DJ stenting, the procedure was successful in 40. Therefore, a total of 60 patients were treated with the antegrade approach, which was successful, with minimal morbidity, in 59 (98%) of those patients. The main causes of failure in the retrograde approach were the inability to catheterize the ureteral meatus due to distortion of the bladder trigone or to pass through the lower segment of the ureter and the inability to visualize the ureteral meatus^**(1)**^.

**Figure 5:**
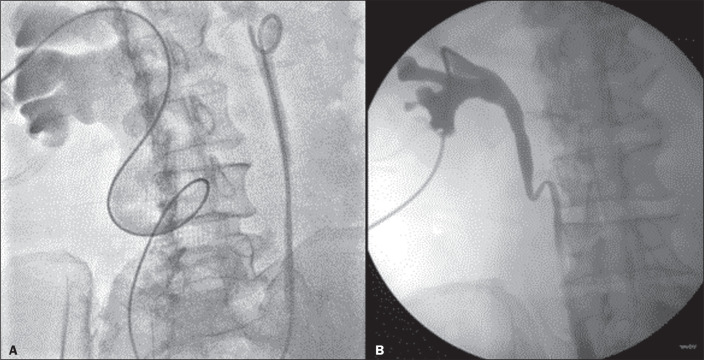
A: Corkscrew ureter on the left and normal ureter on the right. B: Z-shaped ureter.

An extremely important factor for technical success in antegrade DJ stenting is the type of ureteral tortuosity and the extent of ureteral involvement, especially in malignant ureteral obstructions. We note that the unfavorable situations for the antegrade DJ stenting technique are those of a corkscrew ureter (Figure 5) and of a tumor affecting > 5 cm of the ureter.

Complications of antegrade DJ stenting, such as ureteral/vascular injury, formation of an arteriovenous fistula due to vascular injury, and perforation of the artery leading to hemoperitoneum, have been documented in the literature^**(21)**^. However, such complications were not observed in our study sample. The most common complications observed in our sample were dysuria, low back pain, urinary tract infection, and perirenal hematoma.

**Figure 6:**
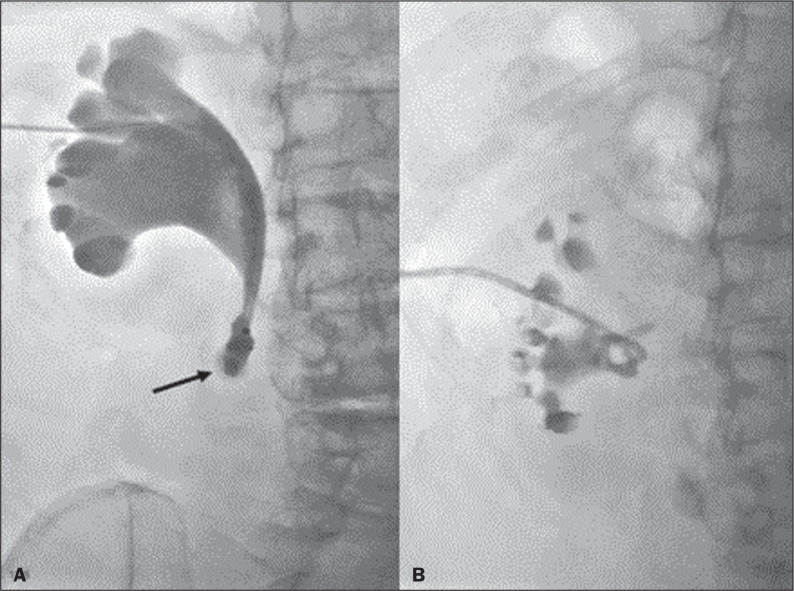
Patient with benign stenosis of the proximal ureter after ureterolithotripsy. Because it was not possible to advance the stent past the point of obstruction (arrow in A), it was necessary to insert a nephrostomy tube and then to perform pyeloplasty by laparoscopy.

In the present study, technical success was achieved in 125 (94.7%) of the 132 procedures performed in patients with malignant neoplasms and in 17 (94.4%) of the 18 performed in patients with non-neoplastic diseases. In one of the patients in the non-neoplastic group, the antegrade approach failed because of accentuated post-ureterolithotripsy fibrosis in the proximal ureter, which made it impossible to advance the stent past the point of obstruction, requiring percutaneous nephrostomy and subsequent ureteral reconstruction via laparoscopy (Figure 6).

Although this was one of the largest studies of antegrade DJ stenting, it was limited by its retrospective nature, which could have resulted in underreporting of clinical conditions. In addition, long-term complications related to DJ stenting, such as bladder irritability, due to irritation by the lower end of the stent, and encrustation of the stent, may not have been reported accurately.

In conclusion, antegrade DJ stenting is a safe, effective method for the treatment of ureteral obstruction, resulting from malignant or benign lesions, when the retrograde (cystoscopic) approach fails.

## CONCLUSION

In the absence of any clinical contraindications and in view of the availability of an interventional radiology team, antegrade DJ stenting can be adopted as a routine approach for the treatment of ureteral obstruction of benign or malignant origin, especially for cases in which the urology team has applied the antegrade approach unsuccessfully.
